# A matched-pair analysis of stereotactic body radiotherapy (SBRT) for oligometastatic lung tumors from colorectal cancer versus early stage non-small cell lung cancer

**DOI:** 10.1186/s12885-018-4865-9

**Published:** 2018-10-10

**Authors:** Xin Wang, Leonid Zamdborg, Hong Ye, Inga S. Grills, Di Yan

**Affiliations:** 1 Department of Abdominal Oncology, Cancer Center, West China Hospital, Sichuan University, No. 37 of Wainan Guoxue Lane, Wuhou District, Chengdu, 610041 Sichuan Province China; 2 Department of Radiation Oncology, Cancer Center, West China Hospital, Sichuan University, Chengdu, China; 3 Department of Radiation Oncology, William Beaumont Hospital, Royal Oak, Michigan USA; 4 Department of Radiation Oncology, Oakland University William Beaumont School of Medicine, Rochester, Michigan USA

**Keywords:** Stereotactic ablative radiation therapy, Oligometastatic tumor, Lung metastasis, Colorectal carcinoma, Dose-response relationship

## Abstract

**Background:**

The use of stereotactic body radiotherapy (SBRT) for early-stage primary non-small cell lung cancer (NSCLC) reported excellent local control rates. But the optimal SBRT dose for oligometastatic lung tumors (OLTs) from colorectal cancer (CRC) has not yet been determined. This study aimed to evaluate whether SBRT to a dose of 48–60 Gy in 4–5 fractions could result in similar local outcomes for OLTs from CRC as compared to early-stage NSCLC, and to examine potential dose-response relationships for OLTs from CRC.

**Methods:**

OLTs from CRC and primary NSCLCs treated with SBRT to 48–60 Gy in 4–5 fractions at a single institution were evaluated, and a matched-pair analysis was performed. Local recurrence-free survival (LRFS) was estimated by the Kaplan-Meier method. Univariate Cox regression was performed to identify significant predictors.

**Results:**

There were 72 lung lesions in 61 patients (24 OLTs from CRC in 15 patients and 48 NSCLCs in 46 patients) were analyzed with a median follow-up of 30 months. LRFS for OLTs from CRC was significantly worse than that of NSCLC when treated with 48–60 Gy/4–5 fx (*p* = 0.006). The 1, 3 and 5-year LRFS of OLTs from CRC vs NSCLC were 80.6% vs. 100%, 68.6% vs. 97.2%, and 68.6% vs. 81.0%, respectively. On univariate analysis, OLTs from CRC treated with higher dose (BED_10_ = 132 Gy) exhibited significantly better local recurrence-free survival than those treated to lower doses (BED_10_ ≤ 105.6 Gy) (*p* = 0.0022). The 1 and 3-year LRFS rates for OLTs treated to a higher dose (BED_10_ = 132 Gy) were 88.9% and 81.5%, vs 33.3%, and not achieved for lower doses (BED_10_ ≤ 105.6 Gy).

**Conclusion:**

The LRFS of OLTs from CRC after SBRT of 48–60 Gy/4–5 fx was significantly worse than that of primary NSCLC. Lower dose SBRT appeared to have inferior control for OLTs of CRC in this cohort. Further studies with larger sample sizes are needed.

## Background

The term “oligometastatic disease” is used to describe a less-advanced state of metastatic disease, limited in the number and sites of metastases, and amenable to potentially curative local therapy [1, 2]. Local treatment may bring the disease under better control through a decrease of the tumor burden, with a consequent improvement in overall survival. Increasingly, local treatment of lung metastases has been accomplished via stereotactic body radiotherapy (SBRT)/stereotactic ablative radiation therapy (SABR) [3, 4].

SBRT/SABR has been rapidly adopted into clinical use in the last decade [5]. Studies that investigated the use of SBRT for early-stage primary non-small cell lung cancer (NSCLC) reported excellent local control rates, typically 83–97.6% at 3 years, with minimal acute or late toxicity [6–12]. Given these results for primary disease, and the possibility of long-term survival in some patients with oligometastases, there has also been increasing interest in using SBRT for the treatment of lung oligometastases [13–20]. Current literature likewise suggests control rates ranging from good to excellent for this application, typically 70–100% with various dose and fractionation schemes [14–23].

Worldwide, colorectal cancer (CRC) is the third-most common cancer in men, and the second-most common in women. It is also the second leading cause of cancer-related death for both sexes combined [24]. Unlike most other cancers, approximately 20% of patients with CRC have metastatic disease at the time of diagnosis, and an additional 20–30% of patients will develop metastases following initial curative resection of the primary tumor [25]. The lung is the second-most frequent site for all colorectal metastases. Nonetheless, cure is still possible for selected stage IV CRC patients, especially for those with oligometastatic liver and/or lung disease. It has been reported that patients with resectable colorectal lung oligometastases have an impressive 5-year overall survival rate of 24–56% following resection [26–30]. Given the encouraging results of SBRT for both metastatic and early stage primary lung tumors, this technique has been used as an effective alternative treatment modality for CRC patients with lung oligometastases, especially those who are not surgical candidates.

While it has been reported that a biologically effective dose (BED) of greater than 100 Gy (assuming an α/β ratio of 10) was needed when performing SBRT for stage I primary NSCLC [31], the optimal dose for oligometastatic lung tumors (OLTs) from CRC has not yet been determined. Dose-fractionation schemes of 48–60 Gy in 4–5 fractions are commonly used for treating stage I primary NSCLC, and result in high local control rates and low toxicities [32–35]. It remains to be seen, however, whether these dose-fractionation schemes can result in similar outcomes for OLTs from CRC. In order to answer this question, and to determine the dose-response relationship for OLTs from CRC, we performed a matched-pair analysis to compare the local recurrence-free survival (LRFS) rate of SBRT to a dose of 48–60 Gy in 4–5 fractions for OLTs from CRC with that of early stage primary NSCLC.

## Methods

### Patient characteristics

Records of patients in the William Beaumont Hospital with primary NSCLC treated with SBRT to a dose of 48–60 Gy in 4–5 fractions on a prospective protocol, as well as those with OLTs from CRC treated with SBRT according to the same dose-fractionation schedules between November 2005 and June 2014 were evaluated. The inclusion criteria for CRC patients were as follows: primary tumor was colorectal adenocarcinoma, one to three biopsy-proven lung metastases ≤5 cm in size, medically inoperable or refusing surgery, and both the primary tumor and any extra-thoracic metastases controlled. Patients with prior lung irradiation, lung surgery, and having chemotherapy, were all eligible. The inclusion criteria for patients receiving SBRT for primary NSCLC were: 1–3 stage I primary histologically-proven NSCLC, and ≤ 5 cm in size. All patients who were not treated with 48–60 Gy in 4–5 fractions were excluded. This study was approved by the institutional review board of William Beaumont Hospital (HIC# 2008–283).

### SBRT technique

The details of SBRT treatment planning and delivery have been described previously [33, 36]. Briefly, all patients were immobilized in a stereotactic body frame (Elekta Oncology, Norcross, Georgia, USA), Alpha Cradle (KGF Enterprises, Chesterfield, Michigan, USA), BodyFIX (Elekta Oncology) or a modified Alpha Cradle/BodyFIX hybrid device. Free-breathing computed tomography (CT) and 4-dimensional CT (Philips Clinical System, Madison, Wisconsin, USA) simulation were performed in all patients. After simulation, acquired CT images were transferred to the planning system (Pinnacle, Philips, Milpitas, California, USA). Pre-treatment PET scans, if available, were fused with the planning CT.

The gross tumor volume (GTV) was defined using CT lung windows and fused PET imaging. The internal target volume (ITV) was composed of the union of the GTV contours on 10 4D phases of respiration. To construct the clinical target volume (CTV), a 3–5 mm margin was applied around the ITV. The planning target volume (PTV) was the CTV plus a 5 mm three-dimensional expansion. A dose of 48–60 Gy in 4–5 fractions was prescribed to the PTV, encompassing the 80% isodose volume (range 60–90%). The prescribed radiation dose must cover ≥95% of the PTV. And 99% of the PTV must receive at least 90% of the prescribed radiation dose. In addition, any dose > 105% of the prescribed dose should not occur outside the PTV. The dose-fractionation schemes were prescribed by the physicians depending on the tumor volume, location, and dose constrains of normal tissues. These dose constrains have been published previously [33, 37]. SBRT plans consisted of 6–9 coplanar and non-coplanar beams with a limited number of couch angles. Intensity modulated radiation therapy was permitted in order to meet normal tissue constraints. Daily online cone-beam CT (CBCT) was performed for soft tissue target registration. Treatment was delivered every other day, with a minimum of 40 h and a maximum of 96 h between fractions. For patients who had multiple metastatic tumors, SBRT for every lesion was delivered sequentially. 4 mg of dexamethasone was administered orally before each fraction.

### Follow-up

Patients on the prospective trial underwent CT and PET/CT imaging to assess tumor response at 6, 16, and 52 weeks following treatment. CT imaging was also performed at 26 weeks following treatment. After 1 year, patients had a chest CT performed every 6 months. Patients with CRC were followed with CT and PET/CT imaging according to a similar schedule, but PET/CT was performed at the physician’s discretion.

Local recurrences were documented either by CT progression consisting of tumor growth after initial shrinkage or after initial stable disease, evident by increase in glucose uptake within the PTV region on FDG-PET scan, or the combination of both, with biopsy confirmation whenever possible. Local recurrences were determined by the treating physician and confirmed with chart and imaging review. Toxicities were graded per the Common Terminology Criteria of Adverse Events Version 3.0 (CTCAE V3.0).

### Statistical analysis

Tumors from the NSCLC and CRC patient cohorts were matched with a 1:2 ratio based on tumor size, tumor location and histology. Because OLTs from CRC typically had a smaller tumor size than NSCLC tumors, the matching criterion was defined as NSCLC tumor size less than or equal to the tumor size of the OLT plus 1 cm. The association of clinical, pathological, and treatment variables within the CRC and NSCLC groups with any given event were analyzed using Student’s t test and Pearson’s Chi-square/Fisher’s exact test. We calculated local recurrence-free survival from the date of SBRT completion to the date of first recurrence or last contact date. For patients who did not have a recurrence but died, we calculated local recurrence-free survival from the date of SBRT completion to the date of death. Patients who did not have a recurrence and did not die were censored at the last follow-up date. Survival was estimated using the Kaplan-Meier method, and compared using the log-rank test. Patient characteristics associated with local recurrence-free survival were identified using univariate Cox regression. All statistical tests were two-sided. A *p* value of less than or equal to 0.05 was considered to be statistically significant. Statistical analyses were conducted with SPSS version 20 (IBM, Somers, New York, USA).

## Results

### Tumor properties

A total of 25 OLTs from CRC and 166 primary NSCLCs were retrieved from the database and met inclusion criteria to undergo matching. A total of 72 lung tumors in 61 patients were matched (Table 1). The number of lung tumors treated per patient was one to three with CRC and one to two with NSCLC. Median tumor size of OLTs from CRC was 1 cm (0.4–1.8 cm), and was 1.55 cm (0.5–2.8 cm) for NSCLC. All patients with NSCLC were stage I. Median follow-up was 30 months (2–69 months) for patients with OLTs from CRC, and 30 months (1–107 months) for patients with NSCLC. There were 3 dose-fractionation schemes used in this study: 60 Gy in 5 fractions (BED_10_ = 132 Gy), 48 Gy in 4 fractions (BED_10_ = 105.6 Gy), and 50 Gy in 5 fractions (BED_10_ = 100 Gy). The NSCLC group had a higher median age, higher percentage of smokers, higher tumor baseline SUV_max_, and more tumors that were treated with 48 Gy in 4 fractions. The CRC group had more patients treated with 60 Gy in 5 fractions.

**Table 1:** Patient characteristics

Characteristics	OLTs from CRC	Early stage NSCLC	p
No. lesions	24	48	
No. patients	15	46	
Age (years)			0.003
Median	62	78	
Range	34–75	49–91	
Gender			0.063
Male	10	18	
Female	5	28	
Smoker			0.019
Smoker	9	41	
Non-smoker	6	5	
Number of lesions per patient			0.0008
1 lesion	9	44	
2 lesions	3	2	
3 lesions	3	0	
Location			1
Central	2	4	
Peripheral	22	44	
Tumor size (cm)			< 0.0001
Median	1	1.55	
Range	0.4–1.8	0.5–2.8	
Pathology			–
Adenocarcinoma	24	48	
Dose			< 0.00001
50 Gy/5 fx	1	0	
48 Gy/4 fx	5	39	
60 Gy/5 fx	18	9	
Baseline SUV_max_			< 0.0001
Median	3.2	5	
Range	1.2–7.7	1.2–18.2	
KRAS-mutation status			
Wild	10	–	
Mutation	11	–	
Unknown	3	–	

### Local recurrence-free survival

**Fig. 1:**
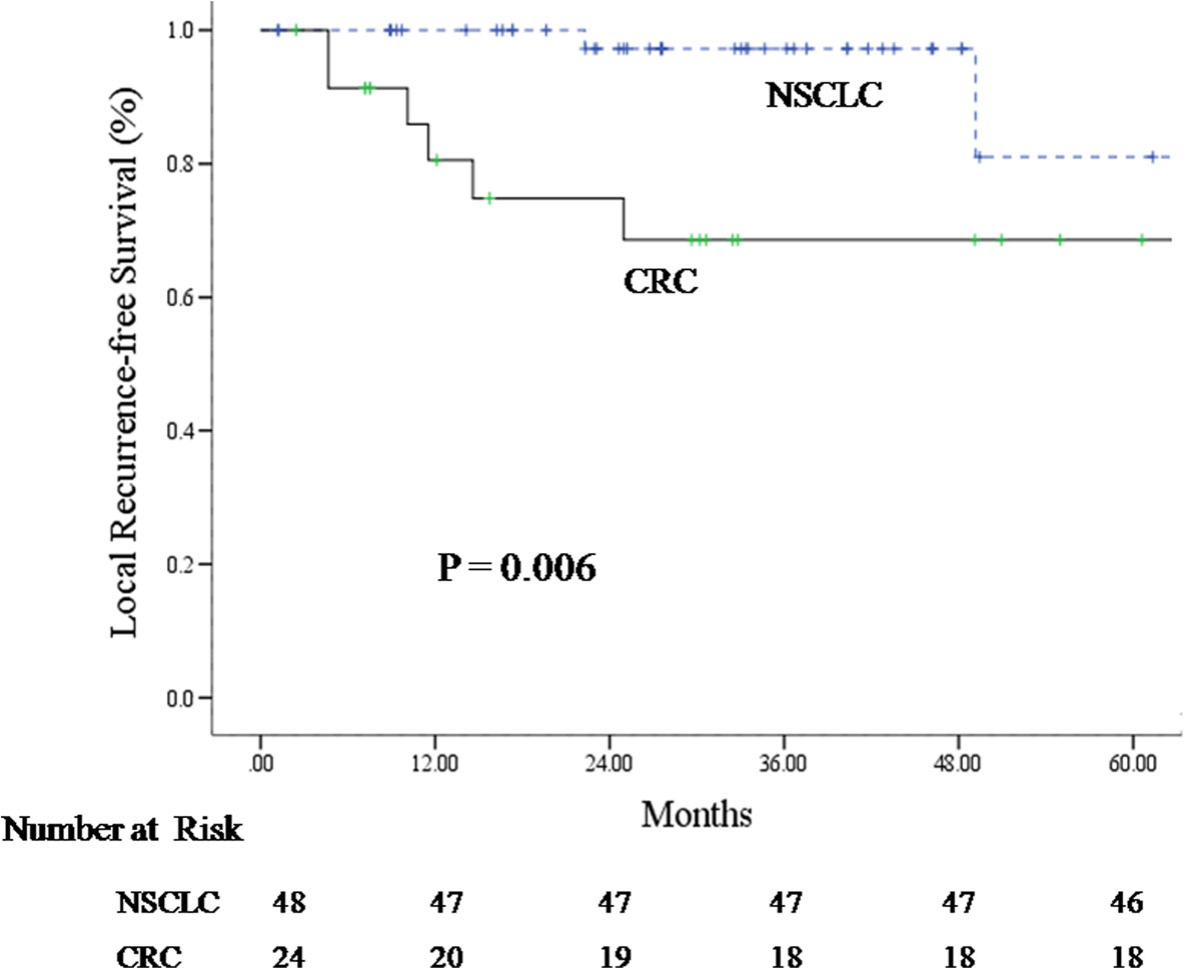
Local recurrence-free survival curve for oligometastatic lung tumors from colorectal cancer and primary NSCLC

At the time of analysis, 6 OLTs (25.0%) and 2 NSCLCs (4.2%) had recurred locally. OLT recurrence was confirmed histologically for one tumor, and was confirmed by PET scan for the remaining 5. One NSCLC recurrence was confirmed by cytology, and the other diagnosed by CT imaging. Local recurrence-free survival significantly favored stage I NSCLC (*p* = 0.006) (Fig. 1). The 1, 3 and 5-year LRFS rates for CRC OLTs and NSCLC were 80.6% (95% confidence interval [CI] 71.8–89.4%) vs 100% (95% CI 100–100%), 68.6% (95% CI 57.7–79.5%) vs 97.2% (95% CI 94.5–99.9%), and 68.6% (95% CI 57.7–79.5%) vs 81.0% (95% CI 66.0–96.0%), respectively.

In univariate analysis (Table 2), the RT dose was significantly associated with local recurrence-free survival for OLTs (*p* = 0.02) but not for NSCLC (*p* = 0.15). The 1 and 3-year LRFS rates for OLTs treated to a higher dose (BED_10_ = 132 Gy) were 88.9% (95% CI 81.5–96.3%) and 81.5% (95% CI 71.7–91.3%), respectively. The 1-year LRFS rate for OLTs treated with lower doses (BED_10_ = 100 Gy or 105.6 Gy) was 33.3% (95% CI 6.1–60.5%), and none achieved 3-year LRFS. Tumor size, baseline SUV_max_, lesion location, and patient age were not significantly associated with local recurrence-free survival for either OLTs or NSCLCs (Table 2). In OLTs, KRAS mutation status likewise did not have a significant impact on local recurrence-free survival. No multivariate analysis was performed.

**Table 2:** Univariate analysis: local control survival for OLTs from CRC and early stage NSCLC

Factors	CRC		NSCLC	
	p	95% CI	p	95% CI
Age	0.31	0.10–1.15	0.85	0.83–1.17
Location	0.34	0.42–1.35	0.34	0.09–2.3
Tumor size	0.72	0.07–6.39	0.95	0.05–23.41
RT dose (BED_10_)	0.02	1.35–53.89	0.15	0.76–5.87
Baseline SUV_max_	0.38	0.42–1.38	0.47	0.82–1.53
KRAS-mutation status	0.23	0.00–16.83	–	

## Discussion

There has recently been growing interest in the use of SBRT for OLTs from CRC. An increasing number of studies have demonstrated that LRFS rates following SBRT differ between OLTs from CRC and primary NSCLCs for a given does-fractionation scheme [18, 38–40]. However, the optimal dose-fractionation schemes for OLTs from CRC remain unclear. In many cases, the same dose-fractionation schemes typically used for early-stage NSCLC (48–60 Gy in 4–5 fractions), are also used for OLTs [37–44]. We thus conducted this retrospective matched-pair comparison of OLTs from CRC and stage I NSCLCs treated with SBRT to a dose of 48–60 Gy in 4–5 fractions to better characterize LRFS of OLTs treated with these common dose-fractionation schemes. All patients with stage I NSCLCs were enrolled on a prospective trial, while all patients with CRC were identified from retrospective review of records. Our results demonstrated that LRFS for OLTs from CRC treated with SBRT to a dose of 48–60 Gy in 4–5 fractions was significantly worse than that of early-stage NSCLC, despite the NSCLC group having a higher percentage of patients that were treated to a lower biologically-effective dose. On univariate analysis, RT dose (BED_10_) was significantly associated with LRFS for OLTs from CRC (*p* = 0.02). A higher dose (60 Gy in 5 fractions, BED_10_ = 132 Gy) resulted in significantly better LRFS of OLTs; however, a BED_10_ ≤ 105.6 Gy appeared insufficient for durable LRFS of OLTs.

Our study was the first matched-pair analysis to compare the outcome of SBRT to a dose of 48–60 Gy in 4–5 fractions for OLTs from CRC and early stage NSCLCs. Recent reports of SBRT for metastatic lung tumors have included limited numbers of CRC patients, ranging from 7 to 65 [27, 37, 39, 40, 43, 45–51]. These studies suggested that local control of metastatic lung tumors from CRC using SBRT was decreased compared to that of primary NSCLCs or metastatic lung tumors from other primary tumors. Kim et al. delivered SBRT to a dose of 39–51 Gy in 3 fractions for 13 metastatic lung tumors from CRC, and reported a three-year local control rate of 52.7% [27]. Takeda et al. [39] analyzed the local control of OLTs resulting from various primary tumors including CRC, and compared them with the local control of primary lung cancer after SBRT to a BED_10_ of 100 Gy in a non-matched fashion. The local control rate in metastatic tumors was significantly worse than that in primary lung cancer (82% vs. 93% at 2 years, *p* <  0.001), and the local control rate of metastases from CRC was significantly worse than that of metastatic tumors of other origins (72% vs. 94% at 2 years, *p* <  0.05). Oh et al. [45] delivered 50–60 Gy in 4–5 fractions to OLTs, and OLTs from the colorectum and liver exhibited a lower local control rate than those from other organs of origin (85.7%, 77.8%, and 100%, *p* = 0.04). Additionally, it was reported in several retrospective studies that recurrent lung tumors treated by SBRT tended to be OLTs from CRC [37, 52, 53]. Baschnagel et al. reported that the 2 year actuarial local failure rate for OLTs from CRC was 20%, versus 0% for all other cases (*p* = 0.001) treated with SBRT (48–60 Gy in 4–5 fractions) [37]. In another retrospective study, Hamamoto et al. [38] delivered 48 Gy in 4 fractions to both stage I primary lung cancer and metastatic lung tumors, and demonstrated 2-year local control rates of 88% and 25%, respectively. They explained that the large proportion of colorectal metastases in their study (7 out of 12, 67%) may be the reason for the poor local control rate for metastatic tumors. They also concluded that local control rates following 48 Gy in 4 fractions were significantly worse in metastatic lung tumors when compared with stage I primary lung cancer, and suggested increasing the SBRT dose for metastatic lung tumors. Several retrospective studies published recently also demonstrated colorectal cancer lung metastases are associated with a higher hazard of local failure and require higher radiation doses [42, 43, 48, 49, 54, 55]. The results of these studies are summarized in Table 3. In our study, the results of local control was similar with the data from the literature. The 1, 3 and 5-year LRFS rates for CRC OLTs in our study were 80.6%, 68. 6% and 68.6%, respectively, which were significant worse than that of NSCLC (100%, 97.2% and 81%, respectively).A number of studies analyzed the dose-response relationship of SBRT for primary lung cancer. It was previously reported that a prescription dose (BED_10_) of 105 Gy or more was correlated with a higher rate of local control for NSCLC (96% vs 85%, *p* < 0.001) [34, 35]. It was also reported that tumor size was associated with the rate of local recurrence [35]. The two-year local recurrence rate of NSCLC with tumors of maximum dimension < 2.0 cm was 2% vs 8% for 2.1–3.0 cm vs 10% for 3.1–5.0 cm (*p* = 0.23) [35]. In our study, all cases of NSCLC were stage I, and our results demonstrated that there were no significant differences in the local control rates between primary NSCLC tumors treated to a higher dose (BED_10_ = 132 Gy) and those treated to a lower dose (BED_10_ ≤ 105.6 Gy).

**Table 3:** Selected series of SBRT for primary NSCLC and/or metastatic lung tumors

Authors	P/M	No. of patients				Dose/fractions	LC		PFS/OS	
		All	P	M	From CRC		P/M	CRC	P/M	CRC
Oh et al. 2012 [45]	M	57	33	24	7	50Gy/5f, 60Gy/5f, 60Gy/4f	3-y: 94.5%	3-y: 80.0%	2-y DFS: 59.7%	
Binkley et al. 2015 [40]	M	77			26	25Gy/1f, 50Gy/4f	1-y: 91.3%; 2-y: 83.8%	1-y: 74.5%; 2-y: 57.8%	2-y OS: 74.7%,	
Agolli et al. 2016 [42]	M				44	23Gy/1f 30Gy/1f 45Gy/3f		1-y: 68.8%; 2-y: 60.2% 3-y: 54.2%		2-y OS: 67.7%; 3-y OS: 50.8% 2-y PFS: 20.3%
Thibault et al. 2014 [44]	P/M	254			45	48-60Gy/4-5f	2-y: 96% (P)	2-y: 76%		
Qiu et al. 2015 [43]	M				65	50Gy/5-10f		2-y: 30%		2-y OS 42.8% 2-y PFS 23.5%
Kinj et al. 2016 [49]	M				53	60Gy/3f		1-y: 79.8% 2-y: 78.2%		1-y OS: 83.8%; 2-y OS: 69.3%
Oskan et al. 2017 [50]	M				40	26-60Gy/1-8f				1-y OS: 84%; 2-y OS: 73% 5-y OS: 39%
Pasqualetti et al. 2017 [54]	M				33	24-27Gy/1f 27-42Gy/3f				Median PFS:13.4 m
Jung et al. 2015 [48]	M				50	40-60Gy/3-4f		1-y: 88.7% 3-y: 70.6%		3-y OS: 64% 3-y PFS: 24%
Hamamoto et al. 2012 [46]	P/M	144	128	31	14	48-60Gy/4-5f	2-y: 87% (P); 2-y: 50% (M)			
Baschnagel et al. 2013 [37]	M	32	0	32	10	48-60Gy/4-5f	1-y: 97%, 2-y: 92%, 3-y: 85%	2-y: 80%	1-y OS:83%, 2-y OS: 76%, 3-y OS-: 63%	
Navarria et al. 2014 [52]	M	76	0	76	29	48Gy/4f, 60Gy/8f, 60Gy/3f	1-y: 95%, 3-y: 89%.	Recurrence in 3 of 29 patients (locally and distance)	1-y OS: 84.1%, 3-y OS: 73%	
Singh et al. 2014 [53]	M	34	0	34	13	50Gy/5f	1-y: 95%, 2-y: 88%, 3-y: 80%.	All 5 patients with local recurrences had CRC		
Kim et al. 2009 [27]	M	13	0		13	39-51Gy/3f		1-y; 76.9%, 2-y: 52.7%, 3-y: 52.7%		1-y OS: 100%, 2-y OS; 75.5%, 3-y OS: 64.7%
Hof et al. 2007 [41]	M	61	0	61	8	13-30Gy/1f	1-y: 88.6%, 2-y: 73.7%, 3-y: 63.1%	3-y: 0%	1-y OS: 78.4%, 2-y OS: 65.1%, 3-y OS: 47.8%	
Nagata et al. 2002 [47]	P/M	40	31	9	4	40-48Gy/4f	1-y: 100% (P); 2-y: 100% (P);	2 mtastases from colon relapsed at 6, 7 m		
Takeda et al. 2011 [39]	P/M	162	113	34	15	50Gy/5f	1-y: 97% (P), 1-y: 86% (M) 2-y: 93% (P), 2-y: 82% (M)	1-y: 80%, 2-y: 72%		
Okunieff et al. 2006 [17]	M	50	0	50		50Gy/10f	1-y: 83%	Had an unexpected number of local failures.		
Yamamoto et al. 2014 [56]	P/M	201	164	37	29	45Gy/3f, 48Gy/4f, 60Gy/8f, 60Gy/15f	3-y: 78%(P), 5-y: 72% (P)	3-y: 26%, 5-y: 26%	3-y OS: 60.9%, 5-y OS: 38.1%	
Helou et al. 2017 [55]	M	120				48-52Gy/4-5f				

*P*: primary NSCLC, *M*: metastatic lung tumor

Unlike primary NSCLC, however, the dose-response relationship for OLTs from CRC has been unclear. Norihisa et al. [39] delivered SBRT to 43 OLTs in 34 patients, of which 9 patients had a colorectal primary. They escalated the RT dose to 60 Gy in 5 fractions after they experienced several local failures with a dose of 48 Gy in 4 fractions. Following this, no local progression was observed in tumors irradiated to a dose of 60 Gy. Rusthoven et al. conducted a phase I/II trial of SBRT for lung metastases which included 9 patients with colorectal primaries [14]. The dose schemes used in this study were 48–60 Gy in 3 fractions (BED_10_ ranged from 124.8 Gy to 180 Gy). Local progression was observed in only one lesion, a metastasis from a sarcoma primary. All colorectal metastases achieved durable local control for the duration of the study. Yamamoto et al. reported that BED_10_ (BED_10_ > 105 Gy vs. BED_10_ ≤ 105 Gy) was a significant independent predictor for local control of primary and metastatic lung tumors [56]. In our study, RT dose (BED_10_ = 132 Gy vs. BED_10_ = 100 Gy/105.6 Gy) was the only statistically significant factor influencing local control rate for OLTs from CRC on multivariate analysis (*p* = 0.028). This suggests that BED_10_ ≤ 105.6Gy is not sufficient for durable local control for OLTs from CRC. Thibault et al. delivered SBRT of 48-60Gy in 4–5 fractions to patients with primary NSCLC and metastatic lung tumors [44]. And it was concluded that covering more of the PTV with the prescription dose was also predictive of higher local control.

The underlying reasons for worse outcomes in OLTs from CRC are unclear. However, based on studies of liver tumors, one may conjecture that metastases from CRC contain larger proportions of hypoxic cells than other tumor types [57], and this hypoxia leads to a decrease in radiosensitivity.

The correlation between KRAS mutation and radiosensitivity for patients with lung metastasis is still unknown. In our study, KRAS mutation status likewise did not have a significant impact on local recurrence-free survival for OLTs from CRC (*p* = 0.23, 95%CI: 0.00–16.83). In a retrospective study, It was reported that KRAS mutation had the correlation with 1-y metastasis-free survival (0% vs. 37.5%; *P* = .04), but not for local control [42].

The role of systematic treatment is unclear for lung OLTs. According to the literature, the most important prognostic factors for lung metastasis SBRT was tumor origin [46, 55, 56]. By ablating OLTs, oncologists might delay the need to start or change systemic treatment [55]. It was reported that the cumulative incidence of change of systemic treatment for patients with single lung metastasis and oligometastases at 12 months were 4.17% and 16.54%, respectively, which were much lower than that of patients with dominant area of progression (50.00%) [55]. It was also demonstrated that both dose escalation and adjuvant chemotherapy might improve local control of SBRT for lung metastasis form CRC [58]. In our study, systematic treatment was not analyzed for the reason of data access. This is one of the limitations of this study. But the efficacies of systematic treatment need further clarification.

The other limitations of this study should also be addressed. This study, though a matched-pair analysis larger than previous series, is nonetheless a single-institution retrospective analysis of a small group of patients. In this study, the comparison were made only between two RT dose schemes, BED_10_ = 132Gy and BED_10_ = 105.6 Gy. Therefore, we could only draw the conclusion based on these two prescription dose. Whether there was an optimal dose between BED_10_ 105.6Gy and 132Gy was still unclear. The most optimal dose schemes need further investigation. And all the results would need to be confirmed in a larger study, preferably a prospective trial.

## Conclusions

In this matched-pair study, the LRFS for OLTs from CRC after SBRT of 48–60 Gy in 4–5 fractions was significantly worse than that of stage I NSCLC. A higher dose (60 Gy in 5 fractions, BED_10_ = 132Gy) resulted in a significantly improved LRFS for OLTs from CRC compared with a lower dose (BED_10_ ≤ 105.6 Gy). Lower dose SBRT appeared to have inferior control for OLTs from CRC in this cohort. Further studies with larger sample sizes are needed, however dose and anticipated local control rates should be considered with discussing SBRT as an option for CRC oligometastases.

## Abbreviations

BED: Biologically effective dose
CBCT: Cone-beam CT
CI: Confidence interval
CRC: Colorectal cancer
CT: Computed tomography
CTCAE: Common Terminology Criteria of Adverse Events
CTV: Clinical target volume
GTV: Gross tumor volume
ITV: Internal target volume
LRFS: Local recurrence-free survival
NSCLC: Non-small cell lung cancer
OLTs: Oligometastatic lung tumors
PTV: Planning target volume
SABR: Stereotactic ablative radiation therapy
SBRT: Stereotactic body radiotherapy
